# Interferon-gamma-induced local leukocytoclastic vasculitis at the subcutaneous injection site*

**DOI:** 10.1590/abd1806-4841.20164985

**Published:** 2016

**Authors:** Fang Wang, Juan-Hua Liu, Yu-Kun Zhao, Di-Qing Luo

**Affiliations:** 1 The First Affiliated Hospital, SunYat-sen University – Guangzhou, China; 2 The Eastern Hospital of The First Affiliated Hospital, Sun Yat-sen University – Guangzhou, China

**Keywords:** Cutaneous, Injection, Interferon-gamma, Leukocytoclastic, Steroid, Vasculitis

## Abstract

Cutaneous reactions associated with interferons (IFNs) treatment are either localized or generalized. The most common presentation of localized reactions at IFNs injection site is usually an erythematous patch or plaque. Local leukocytoclastic vasculitis presenting with cutaneous necrosis is extremely rare. We report a 19-year-old man with hepatitis B who had local leukocytoclastic vasculitis induced by interferon-gama injection at the injection site. After changing the injection sites and using the combined treatment of prednisone and colchicine, the previous lesion healed and no other cutaneous lesion occurred. We also made a mini review of such cases.

## INTRODUCTION

Interferons (IFNs) are a family of five groups of proteins (alpha, beta, gamma, tau, and omega) produced by most cells in response to certain viruses, bacteria, antigens, mitogens, and ribonucleic acid, etc.^1^ Their functions include anti-virus, anti-cellular proliferation, immunoregulation, oncogene inhibition, cellular differentiation, and antiangiogenesis.^1^ Unfortunately, patients receiving IFNs treatment always experience side effects. Common flu-like symptoms and cutaneous reactions have been reported. Occasionally, local skin reactions at the injection site can occur, but skin necrosis is not frequent. Herein, we describe a patient with hepatitis B virus infection who presented with cutaneous necrosis diagnosed as leukocytoclastic vasculitis (LCV) at the injection site of interferon gamma (IFN-γ). The patient showed excellent response to the combined treatment of prednisone and colchicine.

## CASE REPORT

**Figure 1:**
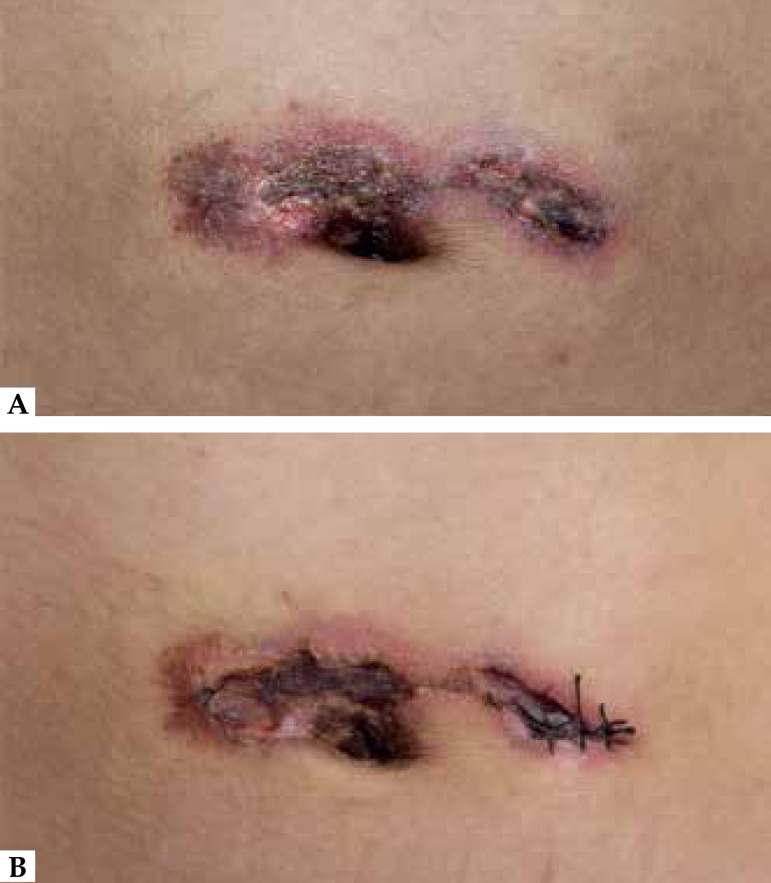
The patient’s skin lesions on the interferon-gamma injection- site. (A) Erythema with central necrosis and exudation localized above the navel before treatment. (B) Rapid improvement presenting as fading erythema and necrosis with crust after a 3-day treatment with prednisone and colchicine

**Figure 2:**
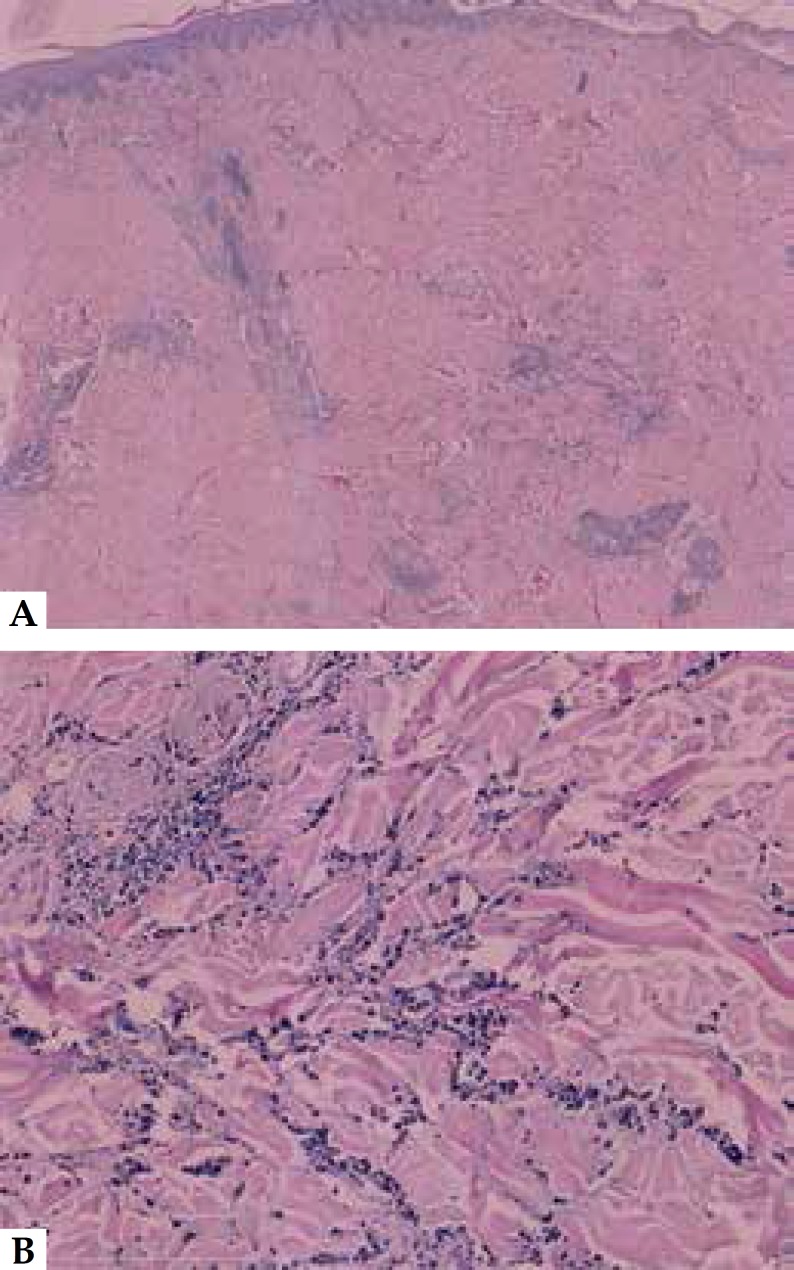
Histopathology of the skin lesion revealed ang iocentric segmental inflammation, endothelial cell swelling and a cellular infiltrate composed of neutrophils showing fragmentation of nuclei (A,B) (HE staining, the length of the white bar is 2 mm and 200 µm for (a) and (B), respectively.)

A 19-year-old man was referred to our clinic with a 3-week history of progressively painful erythema and necrosis on the abdomen. He was diagnosed with hepatitis B virus infection 10 years before. Six months previously, the patient started the treatment with recombinant IFN-γ 6.0×10^7^ units subcutaneously on the abdomen every other day, which resulted in evident decrease of the virus DNA. Three weeks before he came to our institution, painful erythema appeared at the injection site, which progressed rapidly and developed central necrosis subsequently. Repeating injection doses around the lesion could aggravate the pain, the erythema, and the necrosis as well. We observed no other associations. Cutaneous examination revealed erythema with central necrosis and exudation localized above the navel (Figure 1A). Histopathology showed angiocentric segmental inflammation, endothelial cell swelling, and a cellular infiltrate composed of neutrophils showing fragmentation of nuclei (Figure 2). Laboratory tests for complete blood cell count, urine analysis, chemistry profiles, liver function, and auto-immune antibodies were either within normal limits or negative. Eventually, we diagnosed local leukocytoclastic vasculitis (LCV). The patient continued the subcutaneous injection of IFN-γ on the thighs and started with prednisone 30 mg daily and colchicine 0.5 mg twice a day, leading to a rapid improvement after 3 days of treatment (Figure 1B). Colchicine was stopped after 2 weeks of treatment, and prednisone was subsequently tapered off until discontinuation 2 months later. Neither topical nor systemic antibiotics were used. At a 6-month follow-up, the lesion healed leaving behind scars and hyperpigmentation. However, the injection sites on the thighs had neither erythema nor necrosis.

## DISCUSSION

Cutaneous reactions associated with IFN treatment are independent of IFN type and are either localized or generalized, but are not related to the mode of administration (intramuscular or subcutaneous injection) or the site of injection.^1^

The typical clinical presentation of localized reactions at injection sites is usually an ill-defined, pruritic, erythematous patch or plaque, which is generally transient and does not require treatment. Skin ulceration and necrosis seem to be an infrequent complication that occur in less than 4% of individuals receiving interferon beta (IFN-β) and appear to be less frequent with interferon alpha (IFN-α) and IFN-γ.^2^ Rasokat et al.^3^ first described two cases of cutaneous aseptic necrosis after subcutaneous self-injection of recombinant IFN-α in 1989. In 1998, Krainick et al.^4^ summarized 7 cases of patients receiving IFNs therapy, including IFN-α, IFN-β, and IFN-γ, who developed cutaneous necrotizing lesions at the injection sites. They reported that the necrosis was absent for age, sex, or underlying disease predominance, and was usually unrelated to the dose or frequency of administration as well as the injection location. Necrotic ulcers could be single or multiple with various diameters. They mostly developed on the base of an inflammatory plaque that occurred after the onset of treatment varying from weeks to years, usually being months. The major clinical manifestations of our patient who presented with two cutaneous ulcers developing on the base of the erythema were consistent with the previous studies.

The major histopathologic features of a patient with necrotic ulceration at the IFN injection site included perivascular dermatitis with lymphocytic infiltration and thrombosis of deep vessels, and in rare instance, lobular panniculitis.^5^ LCV, as our patient presented, was exceptionally rare. To our knowledge, only 5 cases, including the present one, have been reported (Table 1).^6-9^ Among them, 3 were treated with IFN-α, and the other two were treated with IFN-β and IFN-γ, respectively. The mean age was 46.6 years, with female/ male ratio of 4 to 1. The mean incubation time was 10.2 ± 6.7 weeks. Three cases had non-ulcerative lesions and 2 cases, including our case, developed ulcerative necrosis. Interestingly, the two patients with longer incubation time (12 weeks and 21 weeks, respectively) developed necrosis and ulceration, while the patients with shorter incubation time did not. It is reasonable that violaceous/erythematous papules and plaques are the early manifestations of LCV, whereas necrosis and ulceration are the advanced ones. The symptoms may indicate some clues to diagnosis and treatment.

**Table 1:** Summary of patients with leukocytoclastic vasculitis at injection sites with interferons

Patients					
	1	2	3	4	5
Author	Feldmann et al.^6^	Mary et al.^7^	Pinto et al.^8^	Esra Adisen et al.^9^	The present case
Age (yr)	34	67	56	57	19
Sex	F	F	F	F	M
Indication	Multiple sclerosis	Hepatitis C	Hepatitis C	Hepatitis C	Hepatitis B
IFN type	IFN-β	IFN-α	IFN-α	IFN-α	IFN-γ
Incubation time	12	6	8	4	21
(weeks)					
Locations	Abdomen and thigh	Thigh	Abdomen	Thigh	Abdomen
Lesion number	Multiple	Multiple	Single	Single	Single
Clinical features	Erythema, necrosis	Violaceous/	Violaceous papules	Erythematous plaques	Erythema, necrosis
	and ulceration	erythematous papules			and ulceration
Treatment	Discontinuation of	Discontinuation of	Discontinuation of	Discontinuation of	Combination of pred-
	IFN and systemic	IFN and systemic	IFN and topical corti-	IFN and topical corti-	nisone and colchicine
	prednisolone	prednisone	costeroids	costeroids	

Abbreviations: IFN, interferon; F, female; M, male.

The pathogenesis of cutaneous necrosis caused by IFNs remains unknown. Several possible explanations may be considered. Pre-existing hypercoagulable state in some patients and small vessel endothelial membranes change caused by IFNs may contribute to thrombosis of vessels.^10^ In addition, because IFNs have multiple proinflammatory and immunomodulating activities, such as enhancement of phagocytic activity of macrophages, augmentation of the specific cytotoxicity of lymphocytes for target cells and stimulation of other inflammatory factors^1^, the lesions might be an immunological reaction of the skin vascular. However, the mechanisms of LCV on the IFN injection site still need to be clarified. Perhaps, a type III hypersensitivity reaction is involved.

Cutaneous necrosis is regarded as a severe local adverse effect of IFN treatment. In previous reports, IFN therapy was ceased to resolve the LCV lesions.^6-9^ Other reported treatments included topical care with antibiotics and steroid ointments, surgical debridement and modification of injection site.^6-9^ However, our patient showed excellent response to systemic corticosteroid and colchicine, and no recurrence occurred after change of injection site, suggesting another therapeutic strategy. We considered that IFNs are not forced to be discontinued for the patient with IFN-induced LCV, and low dose systemic corticosteroid and colchicine may be optional for the treatment, especially in early stage of erythema. The physicians should educate the patients regarding as much variation as possible of IFN injection sites to avoid severe local adverse events.
